# P044. Anger expression in chronic daily headache patients with and without psychiatric comorbidity

**DOI:** 10.1186/1129-2377-16-S1-A109

**Published:** 2015-09-28

**Authors:** Giulia Giannini, Marialuisa Rausa, Sabina Cevoli, Valentina Favoni, Rossana Terlizzi, Pietro Cortelli, Giulia Pierangeli

**Affiliations:** Department of Biomedical and NeuroMotor Sciences (DIBINEM) Alma Mater Studiorum, University of Bologna, IRCCS Institute of Neurological Sciences of Bologna, Bologna, Italy

## Background

Previous studies suggest the high prevalence of psychiatric comorbidity in chronic daily headache (CDH) patients. In particular, CDH patients showed higher frequency of anxiety and depressive disorders than episodic migraineurs[1, 2]. However, negative affect emotions (like depression, anxiety and anger) influence the course and impact of headache within the normal range of affective experience, not simply when an Axis I disorder is present[3].

In the literature it is reported that individuals with headache are more likely to hold their anger-in than controls. Individuals who hold anger-in experience an increased pain severity, failure to express anger leads to more disability[4, 5].

Anger levels in headache are supposed to be related to anxiety and depression[6, 7], but one study showed that headache patients hold their anger-in more than controls, even after controlling for depression and anxiety[8].

The aim of this study was to investigate if anger expression levels in CDH patients are related to psychiatric comorbidity.

## Materials and methods

Eighty-five CDH patients (19 M, 72 F) with and without medication overuse were recruited and assessed by Mini International Neuropsychiatric Interview (M.I.N.I.), and State-Trait Anger Expression Inventory (STAXI). On the basis of M.I.N.I. results patients were divided into two groups: with psychiatric comorbidity (group A) and without (group B). STAXI scores were compared between the two groups. T-test was performed to compare continuous variable between groups.

## Results

According to the ICHD-II revised criteria, 4% of subjects had a diagnosis of CM, 19% of CTTH, and 77% of MOH. Psychiatric comorbidity was detected in 39 patients (45.8%) (group A) and was absent in the remaining 46 patients (54.1%) (group B). The disorders most frequently diagnosed were mood and anxiety disorders (43.6%). All STAXI scores were within the normative range, however the highest score was detected in the anger-in subscale, indicating a disposition to suppress rather than express angry feelings. No differences were found between patients with and without psychiatric comorbidity (p = 0.316).

## Conclusions

STAXI results showed no differences in the experience of anger between patients with and without psychiatric comorbidity. Interestingly the highest mean score was in the anger-in subscale that indicates the tendency to suppress anger expression instead of directing it towards other people or objects. Patients with CDH appeared to have a tendency to control their anger expression and to hold their anger-in. The disposition to suppress anger detected in all CDH patients might play a role in the transformation from episodic to chronic headache.

Written informed consent to publication was obtained from the patient(s).
